# ‘Treat everyone like they're a man’: Stakeholder perspectives on the provision of health and social care support for female veterans in the UK

**DOI:** 10.1111/hsc.13790

**Published:** 2022-03-15

**Authors:** Lauren R. Godier‐McBard, Nicola Gillin, Matt J. Fossey

**Affiliations:** ^1^ 2369 Veterans and Families Institute for Military Social Research Anglia Ruskin University Chelmsford UK

**Keywords:** female, gender, healthcare, military, services, support, veterans

## Abstract

International research suggests that female veterans may experience gender‐specific barriers to accessing veteran‐specific care. This is the first UK study to report an exploratory qualitative investigation of the provision of health and social care support for female veterans and whether this support meets their needs. The research team carried out 13 virtual semi‐structured interviews between October and November 2020, with representatives from statutory and third sector organisations that provide support to UK female veterans. Ethical approval was obtained from the Anglia Ruskin University School of Education and Social Care Research Ethics Committee. The authors identified four overarching themes and nine sub‐themes in a thematic analysis following the framework outlined by Braun and Clarke (2006). The findings of this study suggest that practitioners from statutory and third sector organisations perceive the UK veteran support sector as male‐dominated and male‐targeted, with a lack of consideration for female veterans’ needs. Participants reported a lack of engagement with veteran‐specific services by female veterans and suggested that women either do not identify with the ‘veteran’ label or do not feel comfortable accessing male‐dominated veteran‐specific services. The need for specific services for female veterans split participant opinion, with most of those who were female veterans themselves highlighting the importance of ‘safe spaces’ for women, particularly those who had experienced gender‐based violence during military service. Others felt that the veteran support sector currently lacked evidence of women's unique support needs, and an examination of current provision was required. The authors recommend a thorough assessment of UK female veterans’ health and social care needs, alongside development of training and guidance for health and social care professionals, to ensure that veteran services are adequately developed, tailored and targeted with women's needs in mind.

**What is known about this topic?**

- Women are a minority in military veteran communities internationally, with veteran support services designed and built around the needs of men.
- International research suggests that women experience gender‐specific barriers to accessing veteran support services, and underutilise these services compared with men.

**What this paper adds?**

- This is the first UK study to examine whether current health and social care provision for veterans meets the needs of women.
- Findings suggest a perception of UK veteran‐specific support as male‐dominated and male‐targeted, with a lack of engagement from women.
- Health and social care practitioners working within the UK veteran support sector require a better understanding of women veterans’ support needs and how to meet these needs.

## 1 INTRODUCTION

In the UK, a ‘Veteran’ is defined as anyone who has served at least one day in Her Majesty's Armed Forces (Office for Veterans' Affairs, 2020). It is estimated that there are 2.4 million veterans in mainland Britain, of which 11% (approximately 264,000) are women (Ministry of Defence, 2019a). With over 1000 women leaving the Armed Forces each year, this is projected to increase to 13% by 2028 (Ministry of Defence, 2019b). Despite this increase, our understanding of veterans’ health and social care needs in the UK continues to be focused primarily on men (Dodds & Kiernan, 2019).

Once Service Personnel leave the UK Armed Forces, the responsibility for their health and social care falls on civilian statutory (e.g., the National Health Service [NHS]) and third sector (e.g., the charity sector) support services. As such, veterans can access the same health and social care services as UK civilians. However, veteran‐specific support services also exist, including the NHS veteran‐specific mental health services (i.e., ‘Op Courage’ in England; Veterans First Point in Scotland; Veterans’ NHS Wales) (House of Commons Defence Committee, 2019; NHS England, 2021) and the substantial UK military charity sector (Doherty et al., 2019). Indeed, it is suggested that effective health and social care for veterans requires cultural competence, or an understanding of how the military culture may impact on health and well‐being after service (Cooper et al., 2018; House of Commons Defence Committee, 2019).

As women are a minority in the veteran community, support services have historically been designed around the needs of men, leading to male‐focused and male‐dominated veteran‐specific support both in the UK and internationally (Forces in Mind Trust, 2017; Yano et al., 2010). As such, health and social care providers training and experience of working with veterans is likely to be predominantly focused on men's support needs. Furthermore, there is limited research in the UK focused on female veterans’ health and social care support needs and their experiences of accessing support services (Godier‐McBard et al., 2021). The research that does exist primarily focuses on mental health and suggests that female serving personnel and veterans are more likely to seek mental health support than their male counterparts (Iversen et al., 2005; Jones et al., 2019, 2020). However, a preliminary examination of gender differences in help‐seeking in 100 UK veterans suggested that whilst a similar proportion of men and women had accessed mental health support after military service, female veterans were significantly more likely to access *non‐veteran‐specific* NHS support (Godier‐McBard et al., 2022). Furthermore, qualitative findings from this study suggested that women's experiences of stigma and gender‐discrimination in service (i.e., a general perception of female weakness in the military and disparagements around the female gender) had discouraged them from seeking help post‐service. Additionally, female veterans commented on a lack of understanding amongst healthcare professionals of the issues they face following military service and a lack of recognition that women may have experienced the same trauma as men (i.e., combat‐related trauma).

US research also highlights the barriers that women experience in accessing male‐dominated veteran services. These include a lack of gender‐sensitive treatment options (e.g., access to female clinicians and women‐only treatment groups) (Ingelse & Messecar, 2016; Kimerling et al., 2015) and feeling unwelcome in the male‐dominated Veteran's Health Administration (VHA) (Brooks et al., 2016; Kimerling et al., 2015). Furthermore, US female veterans report less satisfaction with veteran‐specific services (Wright et al., 2006) and are shown to underutilise these services compared with men (Thomas et al., 2017). In recognition of these issues, the VHA now operates 1700 facilities offering gender‐specific healthcare services (Congressional Research Service, 2020), which has resulted in increased utilisation of VHA services by women (Vance et al., 2020).

Despite evidence of gender‐specific barriers to care experienced by female veterans, there remains little published UK research focused on the support needs of female veterans. Furthermore, the male‐dominated nature of the veteran‐support sector and preliminary survey evidence suggests that UK health and social care services may not have considered the support needs of women veterans. As such, our aim was to explore the perspectives of practitioners in the UK veteran support sector on the provision of health and social care support for UK female veterans and whether this support meets their needs.

## 2 METHODS

### 2.1 Design

This study utilised an Exploratory Descriptive Qualitative (EDQ) approach (Hunter et al., 2019). The EDQ approach allows researchers to ‘explore and describe the experiences of participants in relation to the phenomena under study’ (p. 5; Hunter et al., 2019). This approach is recommended following an identification of a ‘deficit in knowledge’ (p. 2; Hunter et al., 2019), such as that outlined above. Thirteen semi‐structured interviews were carried out with individuals with experience of providing health and/or social care to UK female veterans. Data were subjected to a thematic analysis (TA) (Braun & Clarke, 2006).

### 2.2 Context

This study formed part of a larger scoping project focused on identifying what is known about the health and well‐being of female veterans in the UK (Godier‐McBard et al., 2021).

### 2.3 Participants

Participants were individuals representing health and social care services (statutory and third sector/charitable organisations) in the UK, which provide support to veterans. Representatives were suggested by the Confederation of Service Charities Female Veteran Cluster, a group established in December 2019, made up of representatives from statutory, third sector and academic organisations, with a vested interest in the issues and challenges faced by female veterans. This group identified participants on the basis that they had experience of providing health and/or social care support to UK female veterans specifically. The purpose of our sampling strategy was to gain insight into whether individuals working within the veteran support services in the UK felt that these services had considered the needs of women and were able to meet these needs. The research team invited 17 participants to take part and 13 individuals consented. Participant gender, veteran status and the type of organisation (i.e., charity or statutory service) represented are presented in Table 1. Nine participants themselves had a military background and most participants were female (10 out of 13). As such, seven participants were female veterans themselves.

**TABLE 1:** Participant gender, veteran status and type of organisation represented

Participant	Gender	Veteran status	Organisation type
1	Female	Non‐veteran	Military charity
2	Female	Non‐veteran	Military charity
3	Female	Veteran	Military charity
4	Female	Veteran	Military charity
5	Male	Veteran	Military charity
6	Male	Non‐veteran	Military charity
7	Female	Veteran	Military charity
8	Female	Veteran	Statutory healthcare service
9	Female	Veteran	Statutory healthcare service
10	Female	Veteran	Military charity
11	Female	Non‐veteran	Military charity
12	Male	Veteran	Statutory healthcare service
13	Female	Veteran	Military charity

### 2.4 Ethical considerations

This project was granted ethical approval for this project by the Anglia Ruskin University School of Education and Social Care Research Ethics Committee (ref: ESC‐SREP‐20‐003). A member of the research team provided participants with information about the project via email at least 24 hours in advance of the interview and gave them the opportunity to ask any questions that they had about the project. Participants were then asked to provide written consent remotely, via DocuSign, and this was reconfirmed verbally prior to commencement of the virtual interview.

### 2.5 Data collection

The research team chose semi‐structured interviews for data collection to allow for a ‘focused’ exploration of the ‘who, what and where’ of the experience (p. 5; Hunter et al., 2019). Due to the restrictions associated with the Covid‐19 pandemic, interviews were carried out virtually via Zoom in October–November 2020 by two members of the research team. One researcher had prior experience of research with female veterans, whilst the other had no experience in this area, but prior experience of conducting qualitative interviews. The interviews lasted between 30 and 60 min. The researchers asked participants the following questions in relation to the support needs and provision for UK female veterans:

1. How well are women veterans in the UK supported by current health and social care service provision for veterans?
2. Where do you think there are gaps in support provision for female veterans in the UK?
3. How do you think efforts to supporting female veterans should be directed?
4. Are there any examples of good practice in supporting female veterans that you would like to share?

Questions were kept purposely broad, and participants were encouraged to discuss anything they felt was relevant to the topic of investigation.

### 2.6 Data analysis

The research team transcribed interviews verbatim and uploaded them into NVivo 12 for analysis. The data were subject to a TA, following the framework outlined by Braun and Clarke (2006). TA is recommended for EDQ studies to identify key issues or the ‘core of the experience’ (p. 6; Hunter et al., 2019). The first step of TA involves familiarisation with the data, which was carried out during both transcription and re‐reading of the transcripts. Second, initial line‐by‐line codes were generated in NVivo 12 by two researchers separately. Once both researchers had reviewed their initial codes, the codes were discussed and compared. Both researchers agreed on the overarching themes and sub‐themes and then sorted the initial codes within these themes. Themes were then refined into a final coding system and re‐applied to the data.

### 2.7 Rigour

The researchers aimed to ensure the trustworthiness (Cypress, 2017) of the findings by using a varied cross‐section of participants working within different organisations that support UK veterans. The questions asked in the interviews were kept broad, to enable participants to discuss what they felt were the key issues, and themes identified in the analysis represent those that occurred across the sample. During data analysis, two researchers coded the transcripts and then an interactive and reflexive process of comparing and refining the themes was carried out, resulting in agreement between the researchers of the overarching themes and sub‐themes that formed the final coding system.

## 3 FINDINGS

The TA of the qualitative interview data led to identification of four overarching themes and nine sub‐themes describing the perspectives of participants working within organisations that provide health and social care services to veterans, of the provision of support for UK female veterans and whether this provision meets their needs. These themes are outlined in the sections below and are summarised in Table 2.

**TABLE 2:** Summary of themes and subthemes

Overarching theme	Sub‐themes	Description
1. Women's needs not considered	*‘Treat everyone like they're a man’*	Participants acknowledged that veteran support services have been developed around men, due to the minority status of women. It was felt that within these support services, women were often provided with support packages that had been designed around men's needs, rather than considering their specific needs.
	*‘Dismissed and unheard’*	Participants reported that women felt invisible within the veteran community and that their needs were not being recognised or met.
2. Male‐dominated nature of the veteran support sector	*‘The charity sector is run by white blokes’*	Participants emphasised that male‐driven nature of the veteran support sector, which was seen to reflect the male‐dominated military culture. This was seen to act as a barrier to recognition of the unique support needs of women.
	*‘Women don't come through the doors’*	Participants also emphasised the fact that beneficiaries within veteran support services were predominantly male. This was seen by participants to discourage women's participation, as they didn't feel services were targeted towards them.
3. Gender‐specific/sensitive services	*The need for ‘Female‐friendly services’*	Some participants felt that support services tailored specifically for women veterans needs were necessary, particularly providing ‘safe spaces’ for women who do not feel comfortable accessing male‐dominated support services. This also included calls for female‐specific peer support and role models in the veteran community.
	*‘Against the spirit of more enlightened times’*	Some participants felt that there was no need for gender‐specific services tailored for women veterans, and that an individualised approach should be taken to providing support, regardless of gender.
	*The ‘un‐evidenced head of expectation’*	Participants felt that that some veteran support services had been developed without an evidence base, and without looking outside of the sector for best practice, leading to the magnification of un‐evidenced support needs. As a result, the importance of ensuring that women veterans support needs cannot already be met within current veteran and civilian services was emphasised, to avoid duplication of services.
4. Increasing awareness and improving training	*‘Deeper knowledge of who women are’*	The need for better understanding and awareness of women veteran's unique experiences and support needs in the veteran support sector was emphasised by participants, including training for health and social care professionals.
	*‘If you don't get to women, then women won't join’*	Participants also felt that veteran support services could do more to increase awareness and communicate their services to women veterans.

### 3.1 Theme 1: Women's needs are not considered

Participants felt that the development of veteran support services in the UK had not historically considered the needs of women, due to their minority status within the veteran community. This was seen as a legacy issue stemming from the discrimination and lack of consideration women had experienced in the military historically.

#### 3.1.1 Sub‐theme: ‘Treat everyone like they're a man’

Participants highlighted a tendency in the veteran support sector to assume parity of treatment across genders, which resulted in a lack of recognition that women may have unique needs:

*“I think what people would say is that “we're fair to everyone”, not realising in saying “we're fair to everyone”, what they mean is “we'll treat everyone like they're a man …” P1, female, non‐veteran, military charity*.

Participants felt that this had led to support packages that were designed for and targeted towards men. This included the transition support provided to service leavers, and health and social care support post‐discharge:

*“When a woman leaves service, everything seems to be male‐dominated … If you look at the career transition workshops, for example, all of the employment opportunities are stereotypically male opportunities*.*” P11, female, non‐veteran, military charity*.

*“We fund a welcome pack for a hostel, which is in a sense a pack of clothes, shower gel, all those types of things. And it’s an interesting question … is there a pack for a female? Does it have a pack of tampons in there? And I bet it doesn’t.” P5, male, veteran, military charity*.

#### 3.1.2 Sub‐theme: ‘Dismissed and unheard’

As a result of the lack of consideration of women's needs, participants reported that female veterans often felt invisible and ignored in the veteran support sector:

*“So, they feel really dismissed and unheard. They feel invisible, they feel like their needs aren't being met and that … the fact that they're a woman isn't celebrated and that their unique differences aren't taken on board at all.” P11, female, non‐veteran, military charity*.

### 3.2 Theme 2: The male‐dominated nature of the veteran‐support sector

Participants emphasised the male‐dominated nature of both the leadership and engagement of beneficiaries in the veteran‐support sector.

#### 3.2.1 Sub‐theme: ‘The charity sector is run by white blokes’

There was a perception amongst participants that the leadership in the veteran charity sector was made up of a narrow demographic of white men from previous service eras (i.e., those who served pre‐2000s), with participants commenting on the lack of women and ethnic minorities in these senior positions:

*“[Discussing a military charity executive] She's probably one of only two charity cheif executives who are female …. And there is certainly nobody from the BAME [Black, Asian and Minority Ethnic] community …” P9, female, veteran, statutory healthcare service*.

Participants felt that this had impacted on the culture of the veteran support sector, leading it to be very ‘male‐driven’ (P1, female, non‐veteran, military charity). Participants described how some men in senior positions in the sector had been in leadership roles in the military several decades ago, and as a result some had brought with them historic discriminatory attitudes towards women:

*“If we've got men in charge and leading our military charities who were of an era, you know, the sixties, the seventies, the eighties, the early nineties who are now in charge of military charities and haven't moved their thinking on. When female veterans come into their orbit, they're still thinking behaving the same way.” P8, female, veteran, statutory healthcare service*.

Participants felt that an understanding of women's needs would be frustrated in the veteran support sector until the lack of female representation in senior positions was rectified.

#### 3.2.2 Sub‐theme: ‘Women don't come through the doors’

Participants saw the male‐driven nature of the veteran support sector as a key factor impacting on engagement and discussed several reasons why this might be discouraging women from accessing veteran‐specific support.

At a fundamental level, it was felt by participants that some women may not identify with the ‘veteran’ label, which they felt was often perceived to relate to men and to those who had been in combat:

*“People in the military have all sorts of different ideas of what a veteran is, and for some of them … a veteran is a soldier who's been fighting … if that's the perception of people from within the military and all the services are saying, “we support veterans”, they're not going to go to this service …” P12, male, veteran, statutory healthcare service*.

As a result, women may not even consider accessing these services, or may question their eligibility.

The very fact that veteran‐specific services are being accessed predominantly by men was also discussed as a potential barrier for women. Participants felt that women may feel unwelcome in these environments, based on prior experiences during their service:

*“I think women still feel in a way that they wouldn't be welcomed. They'd often be the only female, that's the trouble, there'd often be one female amongst 60 blokes. And clearly that's not a very welcoming place to be … Because again, a lot of them come from this culture where they were ridiculed on a daily basis just for their gender.” P3, female, veteran, military charity*.

Furthermore, for some women who had experienced trauma in the form of male violence or abuse against them during service, accessing a male‐dominated support service was seen as a ‘big no‐no’ (P11, female, non‐veteran, military charity), as they may not feel safe and supported in this environment.

### 3.3 Theme 3: The need for gender‐specific/sensitive services

Participant perspectives were split on the need for gender‐specific/sensitive services in the veteran community, with many emphasising their importance, and others highlighting why this might not be necessary to meet women's needs.

#### 3.3.1 Sub‐theme: ‘Female‐friendly services’

Most participants felt that there was a need for tailored support services for female veterans. The importance of women feeling safe and comfortable in support environments was emphasised, particularly for women who had experienced trauma within the male‐dominated military environment:

*“I think for female veterans, there is something around creating what are called psychological safe spaces, whereby groups of women can come together and talk about their experiences. There's a real emergence of people feeling as though they can now start to talk about some of their military sexual trauma … So, I do think we need some safe spaces.” P8, female, veteran, statutory healthcare service*.

To promote women's comfort in support environments, access to female health and social care professionals was seen as imperative. This extended to increased female role models and peer support within the veteran support sector, to provide female veterans with guidance from individuals who understand their gender‐related experiences during and after military service.

*“For those of us that served [and] transitioned successfully and effectively, I think we've got a role to play in helping other female veterans’ transition.” P8, female, veteran, statutory healthcare service*.

#### 3.3.2 Sub‐theme: ‘Against the spirit of more enlightened times’

In contrast, other participants felt that there was not a need for gender‐specific services, and that not all female veterans would be happy with this approach. In conflict with the perspectives provided above, one participant felt that women veterans were accustomed to the male‐dominated military environment:

*“You've got to understand that [for] an awful lot of women who are integrated into military service, the thought about going to something that's all females can be completely against what they're used to … If I said that to my female colleagues in service now, we're going to have set up an all‐women’s group about X, Y, and Z. I would get probably more of a negative reaction than I would a positive reaction because women don't perceive themselves that way.” P13, female, veteran, military charity*.

Additionally, it was felt that this approach conflicted with ensuring equality of treatment for both genders following transition:

*“I think that would probably be against the spirit of more enlightened times to be honest with you … I think there should be a more comprehensive service for all genders in terms of when they leave the armed forces … I don't necessarily see it as a gender thing.” P6, male, non‐veteran, military charity*.

Furthermore, some participants didn't feel that female veterans’ needs were significantly different from those of male veterans. Indeed, some participants felt it was not beneficial to assume the needs of individuals based on their membership of a minority group, and that a more individualised approach should be taken:

*“I've gotta be utterly honest from the casework that we see, there isn't anything that stands out. But I think that goes back to again, that because we don't judge on who we're dealing with, it's almost an irrelevance to us.” P5, male, veteran, military charity*.

*“We're trying to make sure that we don't just look at male and female in terms of gender … What I would say that a service like ours is absolutely blind to how you present. I mean, it's about always helping the individual … So, we don't blindly just apply services. We make sure we understand the individual … It's not about providing [a] female service.” P13, female, veteran, military charity*.

#### 3.3.3 Sub‐theme: The ‘unevidenced head of expectation’

Participants raised concerns that some support services for female veterans had been developed without an evidence base or an assessment of whether the service was required. One participant felt that this could lead to an over‐emphasis of gender differences in support needs, before the sector has clear evidence that these exist:

*“I’m aware that there are some specific charities that are dealing with female‐only sets of issues. Again, my concern is that that creates an un‐evidenced head of expectation, where people will magnify the differences that may or may not exist.” P12, male, veteran, statutory healthcare service*.

Participants felt that there was a tendency within the veteran support sector not to look outside of the sector for best practice, creating an ‘echo chamber’ (P1, female, veteran, military charity sector), in which veteran organisations only consult each other, and duplication of support services often occurs. It was recommended that a thorough assessment be undertaken of whether female veterans’ needs can be met by existing civilian support services, before commissioning additional services:

*“I don’t think we’ve articulated very clearly what that need is and whether there are current services that already exist that could be utilised … To have other healthcare organisations in the military charity sector, many of whom are unproven, trying to provide competitive services. I think we should let the health service do that …. If sexual assault services exist already for women who are sexually traumatised, why would we need a military trauma service?” P9, female, veteran, statutory healthcare service*.

### 3.4 Theme 4: Increasing awareness and improving training

The need to provide appropriate training and awareness of female veterans’ support needs to health and social care professionals, and to promote awareness of services to female veterans was emphasised by participants.

#### 3.4.1 Sub‐theme: ‘Deeper knowledge of who women are’

Participants felt that a better awareness of female veterans’ unique support needs was required in the veteran support sector. It was suggested that this could be achieved with targeted training for health and social care professionals working with veterans, and that this training should seek to develop an understanding of both the unique gender‐related experiences women may have in service (e.g., gender‐based violence), but also recognition that women will often have the same military experiences as men (i.e., combat and deployment):

*“So military sexual trauma is very different to sexual trauma … the staff have to understand there's a big difference. So, I think it's about skilling staff that's already there.” P11, female, non‐veteran, military charity*.

*“If you're a GP and you're trying to understand an issue for a young female sitting in front of you … It's hard to, it'd be easier to say if you've got a young bloke there with a couple of tattoos that say Afghanistan and Iraq and 3 Para … you would immediately go down the line that says, “Oh, did you serve in Afghanistan?” Understanding what that might [mean] in a female context might be slightly difficult to draw out for those professionals. That's to do with professional training and understanding of the context.” P13, male, veteran, statutory healthcare service*.

#### 3.4.2 Sub‐theme: ‘If you don't get to women, then women won't join’

Participants felt that existing veteran support services could do more to promote awareness of their services to female veterans, to ensure they understand that they are eligible to access these services:

*“It's making sure that females … they need to understand what the pathways are, what support mechanisms and organisations are there for them … it's highlighting and better publicising the support mechanisms and the pathways that are available for female veterans.” P4, female, veteran, military charity*.

It was suggested that positive communication from veteran organisations could help to encourage more women to engage with, and feel comfortable utilising veteran‐specific services. This included clearer signposting to available services, but also communication strategies that were more clearly targeted towards women:

*“It needs to be a much more positive communication from the organisations, the national organisations … That they are looking for women members … because if you don't get to women, then women won't join.” P4, female, veteran, military charity*.

*“I think there needs to be a better communication strategy with women … You know, just showing a picture of a female veteran. It's always a white male. Always. It might be an old white male or a young white male, but it's always a white male. And I just think it would make women feel more included”. P3, female, veteran, military charity*.

## 4 DISCUSSION

This project is the first in the UK to qualitatively examine whether the provision of health and social care support for female veterans is meeting their needs. We present a thematic analysis of interview data from 13 participants working in statutory or third sector veteran support organisations, which suggests a perception of the UK veteran support sector as male‐dominated and male‐targeted, with a lack of consideration for female veterans’ needs.

Participants felt that organisations providing support to veterans tended to assume they treated both genders equally, which led to a lack of recognition that women may have unique or unmet needs, and packages of support that had not be developed with women in mind. Indeed, by nature of women's minority status in veteran populations, support services have been built around the needs of male veterans internationally (Forces in Mind Trust, 2017; Yano et al., 2010). Whilst this is unlikely to be intentional, there has yet to be consideration of whether these services adequately engage and meet women's needs in the UK (Godier‐McBard et al., 2021). Indeed, research carried out within US veteran‐specific healthcare services reports that women are less satisfied with these services than men (Wright et al., 2006), and that they are not meeting women's needs (Kehle‐Forbes et al., 2017; Kimerling et al., 2015).

Participants suggested that the lack of focus on female veterans’ needs was not helped by the male‐driven nature of the veteran support sector, of which senior leadership was dominated by males from previous generations of veterans (i.e., those who served pre‐2000s) who do not necessarily understand or represent the experiences of women leaving service in the present day. As a result, female veterans feel unheard and unconsidered regarding their health and social care support needs. Indeed, recent UK research suggests that female veterans feel that their needs are misunderstood or unrecognised by health and social care professionals (Godier‐McBard et al., 2022; Jones, 2018).

Furthermore, participants reported that women were not engaging with veteran‐specific services, supporting emerging evidence of gender differences in engagement with UK veteran‐specific mental health services (Godier‐McBard et al., 2022). Participants suggested multiple reasons for this. Firstly, there was a perception that women did not necessarily identity with the ‘veteran’ label, to which these services were targeted. Indeed, ex‐servicewomen in the UK are less likely to identify as ‘veterans’ compared with their male counterparts (Burdett et al., 2013), and US research links this to reduced utilisation of veteran‐specific healthcare services (Di Leone et al., 2016). Secondly, the beneficiaries of veteran‐specific services are predominantly men, and this was seen as a potential barrier to women, making them feel less comfortable in the support environment. Male‐dominated veteran‐specific services were described as a ‘big no‐no’ for those women who had experienced gender‐based violence and trauma during military service. This is in line with US research which suggests that women with a history of military sexual assault are more likely to perceive veteran healthcare services as unwelcoming and uncomfortable due to being ‘surrounded by men’ (Kehle‐Forbes et al., 2017).

Despite this, participants were split on the need for gender‐specific services. Some argued these were imperative to ensure safe and comfortable environments for women to receive treatment. Others felt that this was not something that would be welcomed by all female veterans, who were used to being in a male‐dominated environment, and that this conflicted with ensuring equality of support for both genders. Interestingly, although most themes were supported by the perspectives of both male and female participants, findings in relation to the need for gender‐specific services appear to be related to participant gender. Indeed, all participants who endorsed the need for ‘female‐friendly services’ were female veterans themselves. Conversely, male participants who commented on this issue indicated that they did not feel that women veterans had unique support needs and that having female‐specific services conflicted with providing parity of treatment. This is perhaps unsurprising, as those with lived experience of the issues and challenges faced by female veterans may be more likely to perceive these as unique and requiring tailored support. However, this highlights the importance of involving those with lived experience in the development and commissioning of services.

Furthermore, the diversity amongst participants in terms of their preferences for gender‐specific services serves to highlight the importance of individual choice and person‐specific care provisions. Indeed, some civilian women would prefer not to attend mixed gender services (Women's Resource Centre, 2006), opting for female‐only spaces due to the perceived physical and psychological safety they can provide (Lewis et al., 2015). Services revolving around a single aspect of identity (gender or veteran status) may be off‐putting to some. Nonetheless, there was agreement that some services, i.e., sexual assault services, should be gender‐specific, reflecting wider opinions on the safety critical aspect of female‐only spaces for those who have experienced male violence (Corry, 2018). Furthermore, most participants agreed that there was a clear need for a better understanding of female veterans’ support needs by health and social care professionals working with the veteran support sector to ensure that services are adequately meeting their needs.

### 4.1 Implications for research, policy and practice

Our findings suggest a lack of awareness and understanding of the support needs of UK female veterans amongst veteran‐specific health and social care services. Indeed, the recent ‘Veterans’ Strategy Action Plan 2022–2024’, published by the UK Office for Veterans’ Affairs in 2022, highlights the need to *“ensure that we celebrate and recognise [female veterans] contribution to the Armed Forces, and make sure the services we deliver to female veterans meet their needs*” (p. 15, Office for Veterans’ Affairs, 2022). To meet the commitments made in this document, consideration of women's health and social care needs in future research, policy and practice is imperative.

Participants highlighted the need for a thorough assessment of female veterans’ health and social care needs and whether current service provision meets these needs. This should include assessment of whether female veterans’ support needs can be met by utilising and tailoring current civilian and/or veteran‐specific services (to avoid unnecessary duplication of services) or through the creation of new gender‐specific services. In the UK, improving personalised care is one of the five major changes that the statutory NHS services seek to implement within the next 5 years, acknowledging that “*one‐size‐fits‐all statutory services have often failed to engage with the people most in need, leading to inequalities in access and outcome”* (p. 12, NHS, 2019). Therefore, for practitioners to meet the needs of female veterans in health and social care services, it is essential that the needs and preferences of the individual are acknowledged, and resources are in place to support this.

Furthermore, our findings suggest that veteran services could do much more to target and communicate their services to women. As such, we recommend that the tailoring and development of services is undertaken by those commissioning veteran services in consultation with female veterans, to ensure these services are adequately targeted and communicated to them. Finally, training and guidance for health and social care professionals should be developed by veteran statutory and third sector organisations to raise awareness of female veterans’ support needs and experiences during and after military service. Both gender sensitivity (Than et al., 2020) and military cultural sensitivity (Brommelsiek et al., 2018) have been found to be significantly improved amongst healthcare providers and students following training. How best to design and deliver this training to UK health and social care professionals requires further exploration.

### 4.2 Limitations

This is the first UK study to examine the support provision provided to female veterans and if this support meets their needs. However, some limitations are of note. Whilst gathering the perspectives of practitioners working with female veterans in the health and social care support sector provides us insight into the experiences of women in accessing support, research collecting data directly from female veterans is needed to confirm our findings. However, seven participants in the sample were female veterans themselves, and as discussed above, their perspectives are likely to have been impacted by their own experiences as veterans. Additionally, as most participants were female (10 out of 13), this is likely to have impacted perspectives on gender‐related issues. However, as evidenced by the quotes outlined in the Findings section, most themes included both a male and female participant perspective. In addition, whilst our sample size was in line with that recommended for EDQ studies, further mixed methods research with a larger sample is needed to evidence the generalisability of the findings beyond the current sample.

## 5 CONCLUSION

Whilst women are a minority in the UK veteran population, their numbers are growing, with over 1000 women leaving the Armed Forces each year. Despite this, our findings highlight a perception of the UK veteran support sector as male‐dominated and male‐targeted, with a lack of consideration of female veterans’ needs. Furthermore, participants reported a lack of engagement with these services by female veterans, who either may not identify with the ‘veteran’ label or may not feel comfortable accessing male‐dominated veteran‐specific services. These findings suggest that female veterans have unique support needs and experiences, and health and social care practitioners working within veteran services should be adequately supported to meet these needs. A thorough assessment of UK female veterans’ health and social care needs is required, alongside development of training and guidance for health and social care professionals, to ensure that veteran‐specific services are adequately developed and tailored with women's needs in mind.

## CONFLICT OF INTEREST

Lauren Godier‐McBard is a member of the Confederation of Service Charities Female Veteran Cluster Group. Nicola Gillin has no conflicts of interest to report. Matt Fossey is co‐chair of the NATO research panel “HFM‐295 RTG Sexual violence in the military.”

## Data Availability

Research data are not shared.
